# A Comparative Study on the Reduction Modes for Quinone to Determine Ubiquinone by HPLC with Luminol Chemiluminescence Detection Based on the Redox Reaction

**DOI:** 10.3390/molecules28010096

**Published:** 2022-12-22

**Authors:** Naoya Kishikawa, Mahmoud El-Maghrabey, Miharu Tobo, Naotaka Kuroda

**Affiliations:** 1 Graduate School of Biomedical Sciences, Course of Pharmaceutical Sciences, Nagasaki University, 1–14 Bunkyo-machi, Nagasaki 852-8521, Japan; 2 Department of Pharmaceutical Analytical Chemistry, Faculty of Pharmacy, Mansoura University, Mansoura 35516, Egypt

**Keywords:** ubiquinone, redox cycle, electrolytic reduction, reduction column, human plasma, chemiluminescence

## Abstract

Ubiquinone (UQ) is considered one of the important biologically active molecules in the human body. Ubiquinone determination in human plasma is important for the investigation of its bioavailability, and also its plasma level is considered an indicator of many illnesses. We have previously developed sensitive and selective chemiluminescence (CL) method for the determination of UQ in human plasma based on its redox cycle with dithiothreitol (DTT) and luminol. However, this method requires an additional pump to deliver DTT as a post-column reagent and has the problems of high DTT consumption and broadening of the UQ peak due to online mixing with DTT. Herein, an HPLC (high-performance liquid chromatography) system equipped with two types of online reduction systems (electrolytic flow cell or platinum catalyst-packed reduction column) that play the role of DTT was constructed to reduce reagent consumption and simplify the system. The newly proposed two methods were carefully optimized and validated, and the analytical performance for UQ determination was compared with that of the conventional DTT method. Among the tested systems, the electrolytic reduction system showed ten times higher sensitivity than the DTT method, with a limit of detection of 3.1 nM. In addition, it showed a better chromatographic performance and the best peak shape with a number of theoretical plates exceeding 6500. Consequently, it was applied to the determination of UQ in healthy human plasma, and it showed good recovery (≥97.9%) and reliable precision (≤6.8%) without any interference from plasma components.

## 1. Introduction

Ubiquinone (UQ, CoQ10) is a fat-soluble molecule that is a component of the mitochondrial electron transfer system. UQ is mainly found in the inner mitochondrial membrane of cells and plays a role in stimulating the biosynthesis of ATP by activating the electron transfer system through the redox cycle [1]. UQ exists in both oxidized (ubiquinone) and reduced (ubiquinol) forms, and the reduced form mainly exhibits antioxidative activities. It has been reported that most of the UQ in blood and tissues exists in the reduced form [2,3,4,5,6]. Reduced UQ binds to lipoproteins and circulates in the body, protecting it from various oxidative stresses such as lipid peroxidation of biomembrane and DNA damage [7,8]. In addition, the amount of UQ in the body decreases with age [9], and also it has been found that the plasma concentration of UQ is significantly decreased in patients with hyperthyroidism [10], melanoma [11], and mevalonic aciduria [12] compared to healthy subjects. It is also known that the ratio of reduced UQ/oxidized UQ is decreased in diabetic patients compared with healthy controls [13]. These changes are considered to be from the decrease in the biosynthesis of UQ and the conversion efficiency to reduced UQ. Since UQ has a wide range of physiological roles and is also useful as a biomarker for understanding human health conditions [14], it would be useful to measure the concentration of UQ in biological samples.

Several methods have been developed to measure UQ, including absorption spectrophotometry [15], microplate-based colorimetric assay [14], photoelectrochemical assay [16], nuclear magnetic resonance spectroscopy (^1^H-NMR) [17], high-performance liquid chromatography-absorption spectrophotometry (HPLC-UV) [18], HPLC-electrochemical detection (ECD) [2,3,19] and tandem mass spectrometry (MS/MS) [6]. However, in general, ^1^H-NMR is not sufficiently sensitive, and absorption spectrophotometry and HPLC-UV are not sufficiently selective in addition to their poor sensitivity. Additionally, the microplate-based colorimetric assay and photoelectrochemical assay suffered from their low selectivity and their non-applicability to analysis of complex matrices such as plasma. The HPLC-ECD method is commonly used for the determination of UQ due to its high sensitivity and selectivity, but it requires a special device called a multi-electrode ECD because UQ needs to be reduced by chemical or electrochemical methods immediately before the measurement. The LC-MS/MS method is highly sensitive and selective but has the disadvantage of requiring complicated, expensive equipment and matrix effects [20]. Fluorimetric (FL) and HPLC-FL methods are usually a good choice for the analysis of xenobiotics [21,22] due to their simplicity, selectivity, and high sensitivity; however, UQ has very low native fluorescence and only in its reduced form [23].

**Figure 1:**
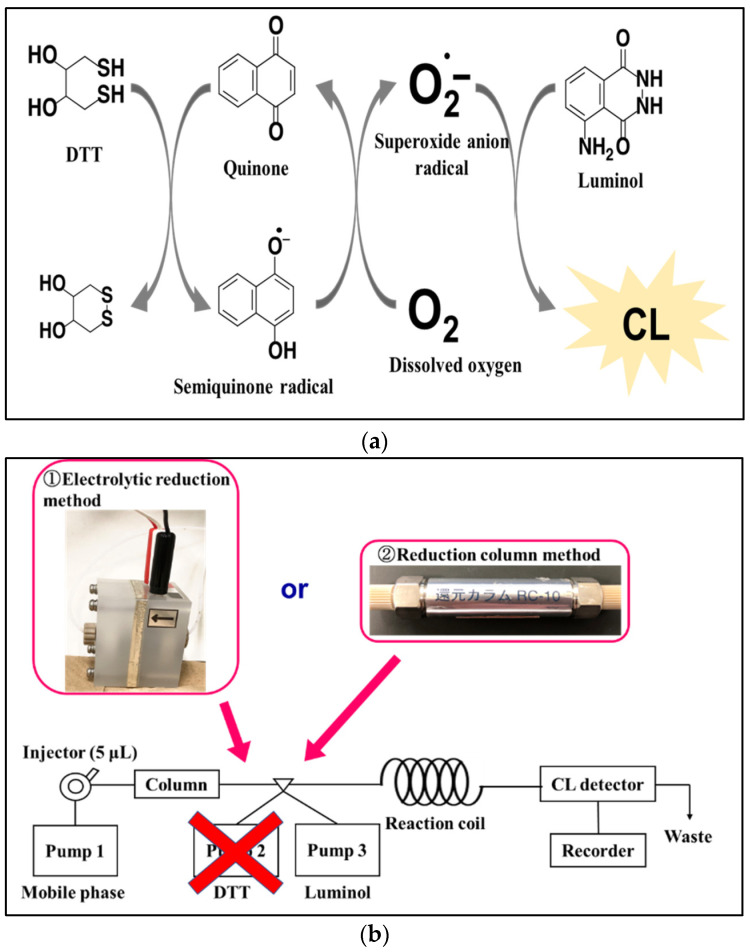
The CL (chemiluminescence) platforms for quinone determination, where (**a**) the mechanism of CL reaction for quinone based on the generation of superoxide anion radical through their redox cycle with DTT (dithiothreitol), and (**b**) HPLC−CL (high-performance liquid chromatography chemiluminescence) system of the proposed methods, including the classical chemical-based reduction using DTT and its replacement with the online electrolytic reduction and reduction column.

In order to overcome these disadvantages, we previously developed sensitive and selective chemiluminescence (CL) method for the determination of various quinones, including UQ, using the redox cycle of quinones [24,25,26,27,28]. Quinone is reduced to unstable semiquinone radicals by reacting with reducing agents such as dithiothreitol (DTT), which react with dissolved oxygen to produce reactive oxygen species (ROS) in the process of re-oxidation. The reaction of these reactive oxygen species with luminol, a CL reagent, yields a CL proportional to the concentration of quinone (Figure 1a). We have established an HPLC-CL method based on this CL reaction system for the determination of UQ contained in plasma samples. While this HPLC-CL method is capable of highly sensitive and selective determination of UQ, it requires an additional pump to deliver DTT as a post-column reagent and has the problems of high DTT consumption and broadening of the UQ peak due to mixing with DTT. In the present study, an HPLC system equipped with two types of online reduction systems (electrolytic flow cell or platinum catalyst-packed reduction column) that play the role of DTT (Figure 1b) was constructed to reduce reagent consumption and simplify the system, and the analytical performance for UQ was compared. Furthermore, the electrolytic reduction system, which showed better sensitivity, was applied for the determination of UQ in human plasma.

## 2. Result and Discussion

### 2.1. Electrolytic Reduction in Ubiquinone

**Figure 2:**
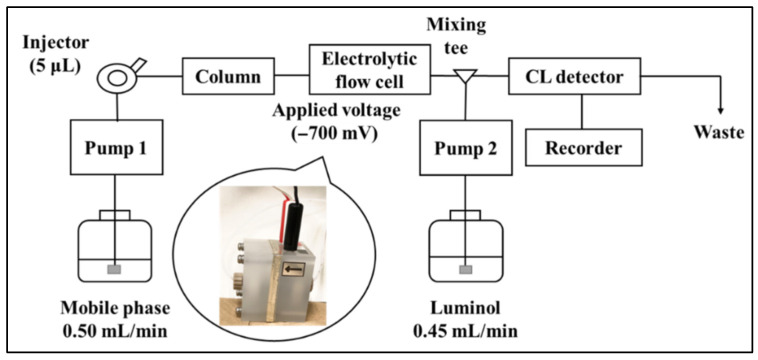
HPLC−CL system employing the electrolytic reduction.

**Figure 3:**
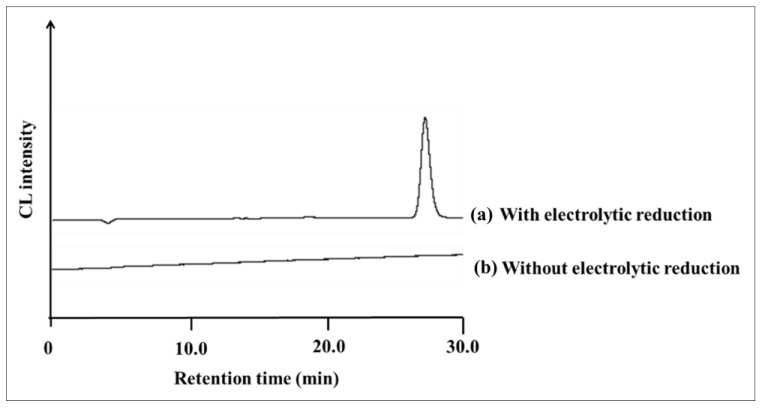
Chromatograms of 1.0 μM UQ standard obtained (**a**) with electrolytic reduction and (**b**) without electrolytic reduction by HPLC-CL detection.

The chromatogram of UQ in the HPLC-electrolytic reduction-CL system was obtained by injecting 1.0 μM (5.0 pmol/injection) of UQ standard solution into the HPLC system shown in Figure 2, and the chromatogram is shown in Figure 3. The peak of UQ was detected at the retention time of 28.1 min in the case of electrolytic reduction. On the other hand, no UQ peak was detected in the absence of electrolytic reduction. Therefore, it is considered that UQ is converted to the semiquinone radical by electrolytic reduction and that CL is generated by the reaction of reactive oxygen species generated in the process of re-oxidation to UQ with luminol mixed online.

**Figure 4:**
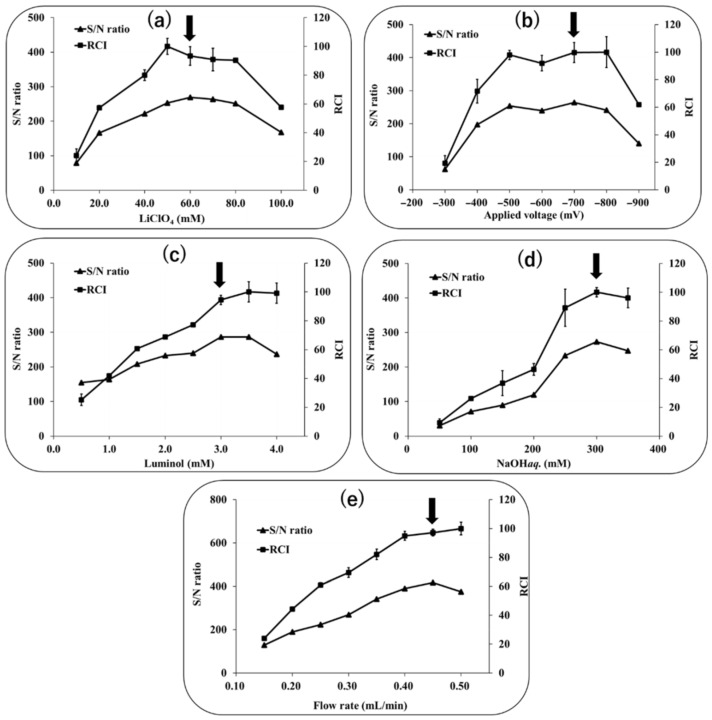
The effects of different factors affecting the online electrolytic reduction—luminol CL system for UQ, including (**a**) LiClO_4_ concentration, (**b**) applied voltage, (**c**) luminol concentration, (**d**) luminol solvent (NaOH*aq*. concentration), and (**e**) luminol flow rate, on relative chemiluminescence intensity (RCI) and signal to noise (S/N) ratio. The arrows indicate S/N ratio maximum.

In order to obtain higher sensitivity, electrolytic reduction and CL conditions were optimized using the standard solution of UQ. According to Wang et al., the commonly used supporting electrolytes for the reduction in quinones are either tetrabutylammonium (TBA)^+^, Li^+^, or H^+^-based electrolytes. Li^+^-based electrolytes are the best as it results in intermediate redox potential, while TBA^+^ and H^+^ result in extremely low and high redox potential, respectively. In addition, bulky TBA^+^, as the cycling ion, makes the stabilization of quinone-reduced stat not possible, and the reduction potential was largely decreased. In addition, in H^+^-based electrolytes, the interaction between the charges is largely affected by the stabilization of the counterion, resulting in a disproportionation of the semiquinone radical intermediate state. From all this, LiClO_4_ is the electrolyte of choice for the electroreduction in quinone. Therefore, it was used in our developed method [29]. For efficient electrolytic reduction, the concentration of the supporting electrolyte is important to increase the electrical conductivity. In the proposed method, the concentration of lithium perchlorate (LiClO_4_) was added to the mobile phase as a supporting electrolyte and was examined in the range of 10.0–100.0 mM (Figure 4a). Both peak height and S/N ratio increased with increasing LiClO_4_ concentration, and the maximum and constant peak height and S/N ratio were obtained between 50.0–80.0 mM. Therefore, 60.0 mM was selected as the optimum condition because it gave the largest S/N ratio. The applied voltage for electrolytic reduction was investigated in the range from −300 to 900 mV. As shown in Figure 4b, both peak height and S/N ratio were maximum at −700 mV. Therefore, −700 mV was selected as the optimum applied voltage. From the literature, increasing temperature leads to a decrease in the electrochemical reduction efficiency [30], and the optimum temperature for the electroreduction in UQ was reported to be room temperature [31]. Hence, the experiment was conducted at room temperature. The luminol concentration was examined in the range of 0.5–4.0 mM. The peak height increased as the luminol concentration increased, and the maximum peak height was obtained at 3.0 mM or higher. Therefore, 3.0 mM was selected as the optimum luminol concentration, which gave the highest S/N ratio (Figure 4c). The concentration of sodium hydroxide (NaOH) used as the solvent of luminol was investigated in the range of 50–350 mM. As shown in Figure 4d, both the peak height and S/N ratio were maximum at 300 mM. Therefore, the optimum concentration of NaOH was set to 300 mM. The flow rate of the luminol solution was examined in the range of 0.15–0.50 mL/min. As shown in Figure 4e, the peak height was almost the highest at a flow rate of 0.40 mL/min or higher, while the S/N ratio was the highest at 0.45 mL/min. Based on these results, the optimum flow rate of the luminol solution was set to 0.45 mL/min, taking the reagent consumption into consideration.

Under the optimum conditions, calibration curves were constructed using the standard solutions of UQ. UQ gave a good linear relationship between concentration and peak height in the range of 0.010–10 μM with a correlation coefficient *r* = 0.995, and the corresponding regression equation was as follows: Y = 7.00 × 10^4^ X + 8.14 × 10^3^, where Y = peak height (μV) and X = concentration of ubiquinone (μM). When the concentration of UQ that gives a peak height three times higher than the noise (S/N = 3) is defined as the lower limit of detection, the value is 3.13 nM (15.7 fmol/injection). The precision of repeated measurements (*n* = 5) within a day and between days was calculated using the concentrations at three points within the calibration curve, and the relative standard deviation (RSD) of the precision within a day was ≤3.4%, and that between days was ≤3.5%, which indicates the good precision of the developed method (Table 1).

### 2.2. Catalytic Reduction in Ubiquinone by Reduction Column

**Figure 5:**
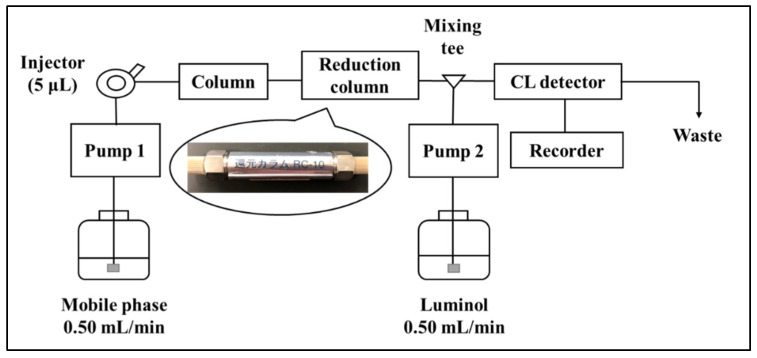
HPLC-CL system employing the reduction column.

**Figure 6:**
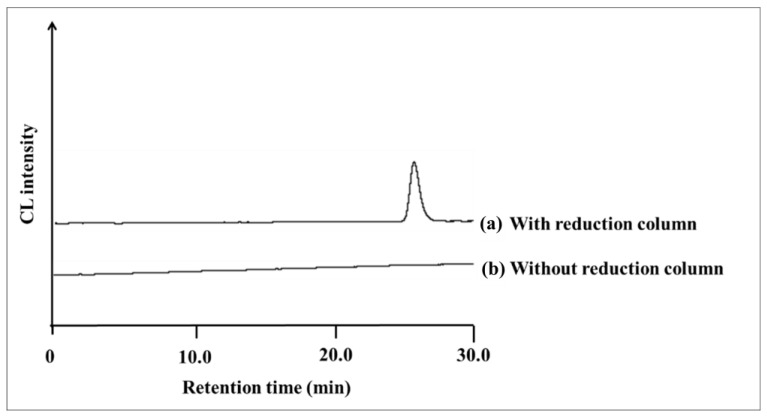
Chromatograms of 1.0 μM UQ standard obtained (**a**) with reduction column and (**b**) without reduction column by HPLC-CL detection.

The chromatogram of UQ in the HPLC-reduction column-CL system was obtained by injecting 1.0 μM (5.0 pmol/injection) of UQ standard solution into the HPLC system shown in Figure 5, and the chromatogram is shown in Figure 6. 

The peak of UQ, which was not detected when the reduction column was not connected, was detected at the retention time of 25.8 min when the reduction column was added online. Therefore, it is considered that UQ is reduced to semiquinone radicals during the passage through the reduction column, resulting in CL.

**Figure 7:**
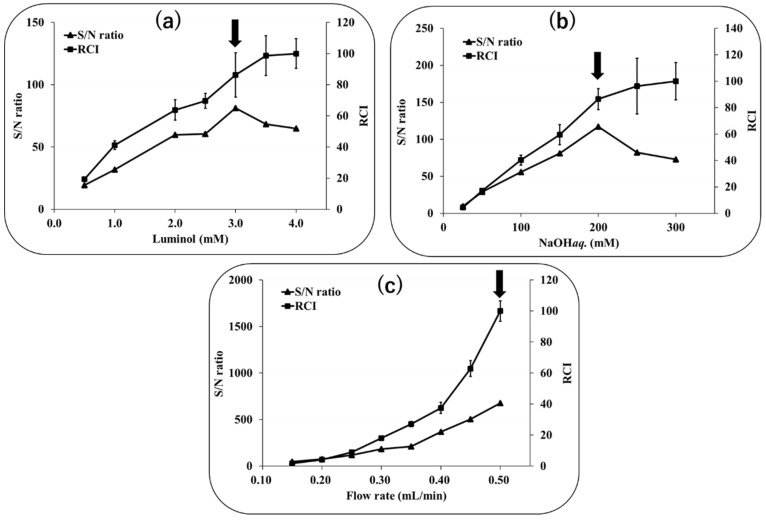
The effects of different factors affecting the online reduction column—luminol CL system for UQ, including (**a**) luminol concentration, (**b**) luminol solvent (NaOH*aq*. concentration), and (**c**) luminol flow rate, on relative chemiluminescence intensity (RCI) and signal to noise (S/N ratio. The arrows indicate S/N ratio maximum.

CL reaction conditions were optimized using the standard solution of UQ. The concentration of luminol was examined in the range of 0.5–4.0 mM. As shown in Figure 7a, the peak height reached its maximum at 3.5 mM, while the S/N ratio reached its maximum at 3.0 mM. Based on these results, the optimum luminol concentration was determined to be 3.0 mM. The concentration of NaOH solution was examined in the range of 25–300 mM. As shown in Figure 7b, the peak height reached a maximum at 300 mM, while the S/N ratio reached a maximum at 200 mM. Based on these results, the optimum concentration of NaOH was determined to be 200 mM. The flow rate of the luminol solution was investigated in the range of 0.15–0.50 mL/min. As shown in Figure 7c, both peak height and S/N ratio tended to increase with increasing flow rate. However, the optimum flow rate of the luminol solution was set to 0.50 mL/min because the consumption of reagents increases at higher flow rates.

Under the optimum conditions, calibration curves were constructed using the standard solutions of UQ. UQ gave a good linear relationship between concentration and peak height in the range of 0.015–10 μM with a correlation coefficient *r* = 0.997, and the corresponding regression equation was as follows: Y = 4.93 × 10^4^ X − 2.59 × 10^3^, where Y = peak height (μV) and X = concentration of ubiquinone (μM). The lower limit of detection was found to be 4.53 nM (22.7 fmol/injection). The precision of repeated measurements (*n* = 5) within a day and between days was ≤2.9% and ≤4.4%, respectively (Table 2), which indicates the acceptable precision of the proposed method

### 2.3. Comparison of the Reduction Modes on the CL of Ubiquinone

Table 3 compares the performance of the electrolytic reduction method and the reduction column method with that of the conventional DTT method [25]. Compared to the conventional DTT method, the detection sensitivity of UQ was 10 times higher for the electrolytic reduction method and 6.7 times higher for the reduction column method. This is probably due to the known higher efficiency of column-based and electrochemical-based reduction than chemical reduction [32,33], and also, reducing agents such as dithiothreitol are usually not highly stable. Additionally, the number of pumps required for the DTT method is three, while that for the electrolytic reduction and reduction column methods is two, thus improving the disadvantages of reagent consumption and the need for additional equipment. Comparing the theoretical number of plates, the theoretical number of plates of the two reduction methods examined in this study was larger than that of the DTT method, suggesting that the problem of diffusion of peak fractions due to mixing and dilution with the reductant solution was overcome. In comparison between the electrolytic reduction method and the reduction column method, the electrolytic reduction method can control the reduction power by changing the voltage applied to the compound, while the reduction column method does not require a supporting electrolyte. From these results, it was demonstrated that the proposed electrolytic and column reduction methods showed better analytical performance than the conventional DTT method. In addition, it was demonstrated that the electrolytic reduction method showed the best sensitivity. Therefore, it was applied in the next experiments for the determination of UQ in human plasma.

### 2.4. Application and Validation of the Proposed Electrolytic Reduction-CL Method for Determination of UQ in Human Serum

**Figure 8:**
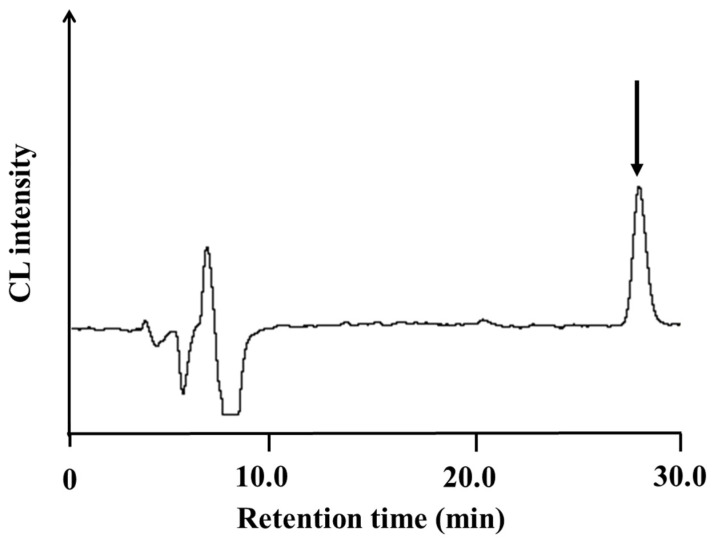
Chromatogram of UQ in human plasma by CL detection after electrolytic reduction. The peak indicated by the arrow corresponds to UQ.

The HPLC-CL system based on the electrolytic reduction method for UQ was used for the determination of UQ in human plasma. The HPLC-CL system enables selective determination of UQ in human plasma without the influence of foreign substances coexisting in the plasma. Plasma samples were extracted with hexane as described in the experimental section and then determined by the proposed method. A calibration curve was constructed using human plasma samples supplemented with UQ, and a good linear relationship between concentration and peak height was obtained in the range of 0.010–2.0 μM, with a detection sensitivity of 3.37 nM (16.9 fmol/injection). The calibration regression equation was as follows: Y = 3.05 × 10^4^ X − 1.56 × 10^3^, where Y = peak height (μV) and X = concentration of UQ (μM). Figure 8 shows the chromatograms of human plasma pretreated according to the procedure in the experimental section. As shown in Figure 8, UQ in non-spiked human plasma could be selectively detected with a retention time of 28.1 min without the influence of coexisting components. The concentration of UQ in the human plasma of five healthy volunteers was determined by the proposed method and was found to be as follows: 0.29, 0.33, 0.36, 0.62, and 0.44 µM, with an average value of 0.41 ± 0.12 μM, which is almost equivalent to the quantitative value determined by the DTT method [25] and previous literature values [2,3,6,18]. The recovery rate of UQ from human plasma was more than 97.9%, and the accuracy of repeated measurements was good (≤6.8%), as shown in Table 4, indicating that this method is useful and reliable for the determination of UQ in biological samples.

The sensitivity of the developed electrolytic reduction-CL method for UQ determination was compared with those of the UQ quantification methods reported so far (Table 5). The sensitivity of the electrolytic reduction-CL method was more than 60 times higher than that of the absorption spectrophotometry [15] and more than 2000 times higher than that of ^1^H-NMR [17]. When the sensitivity per injection volume was compared with that of the HPLC-UV [18], HPLC-ECD [2,3,19], HPLC-DTT-CL [25], and LC-MS/MS [6] methods, all of them were 2.5–21 times higher than that of the sensitivity the developed method. These data demonstrate the superiority of the analytical performance of the developed method over all other reported methods in the literature.

## 3. Experimental

### 3.1. Material and Reagents

UQ, luminol, and NaOH were sourced from Sigma (St. Louis, MO, USA), Merck (Tokyo, Japan), and Kanto Chemical (Tokyo, Japan). DTT, ethanol, and methanol were purchased from Nacalai Tesque (Kyoto, Japan). A Simpli Lab UV (Millipore, Bedford, MA, USA) water device was used to supply purified water. Other chemicals were of extra pure grade. Ethanolic Stock solutions of UQ were prepared and further diluted with ethanol to prepare the working solutions. Solutions of DTT and luminol were prepared in methanol and NaOH aqueous solutions, respectively, just before analysis.

### 3.2. HPLC System with Electrolytic Reduction

The HPLC system (Figure 2) was comprised of two pumps (LC-10AS, Shimadzu, Kyoto, Japan), an injector (Rheodyne 7125, Cotati, CA, USA) with a 5-μL sample loop, a chemiluminescence detector (CLD-10A, Shimadzu, Kyoto, Japan), a noise filter (UNI-1, Union, Gunma, Japan), and a chromatorecorder (SIC, Tokyo, Japan). A Cosmosil 5C8-MS (150 × 4.6 mm, i.d., 5 μm, Nacalai Tesque, Kyoto, Japan) and 60.0 mM LiClO_4_ in methanol were used as stationary and mobile phase, respectively. UQ eluted from the column was introduced into the electrolytic reduction flow cell PEC-510C (Eicom, Tokyo, Japan) and reduced by applying a voltage of −700 mV, then mixed with 300 mM NaOH solution of 3.0 mM luminol and introduced into the CL detector. The flow rates of the mobile phase and luminol solution were set at 0.50 and 0.45 mL/min, respectively.

### 3.3. HPLC with Reduction Column

The HPLC system (Figure 5) was comprised of two pumps (LC-10AS, Shimadzu, Kyoto, Japan), an injector (Rheodyne 7125, Cotati, CA, USA) with a 5-μL sample loop, a chemiluminescence detector (CLD-10A, Shimadzu, Kyoto, Japan), a noise filter (UNI-1, Union, Gunma, Japan), and a chromatorecorder (SIC, Tokyo, Japan). A Cosmosil 5C8-MS (150 × 4.6 mm, i.d., 5 μm, Nacalai Tesque, Kyoto, Japan) and methanol were used as stationary and mobile phases, respectively. UQ eluted from the column was introduced into a platinum-packed reduction column Daiso RC-10 (15 mm × 4.0 mm, i.d.) for reduction, mixed with a 200 mM NaOH solution of 3.0 mM luminol, and introduced into a CL detector for measurement. The flow rates of the mobile phase and luminol solution were set at 0.50 and 0.50 mL/min, respectively.

### 3.4. Assay Procedure for Ubiquinone in Human Serum

Plasma samples with a volume of 50 µL were mixed with 10 μL 0.3% hydrogen peroxide (for oxidation of ubiquinol to UQ). Next, 100 μL of ethanol was added (for protein denaturation) followed by 1 min vortex. To this extraction mixture, *n*-hexane (600 μL) was added. Next, the organic layer was separated and then evaporated under reduced pressure. Afterward, the residue was reconstituted with 50 μL of methanol and then injected (5 μL) into the HPLC system. It is important to mention that The Ethics Committee of the School of Pharmaceutical Sciences, Nagasaki University, has approved the present study (approval number 22) and that the experiment was performed in accordance with established guidelines.

## 4. Conclusions

In this study, we investigated the development of an HPLC-CL quantification system for UQ based on the principle of inducing the redox cycle of quinone by the electrolytic reduction method and the reduction column method, which are alternative techniques to the conventional chemical reduction that uses DTT. After optimizing the reaction conditions for each reduction method, the analytical performance, including sensitivity, was compared with that of the DTT method. The HPLC-CL system based on the electrolytic reduction method showed the highest sensitivity and the best peak shape. Thus, it is the recommended one for the sensitive analysis of UQ. Consequently, it was applied to the determination of UQ in human plasma, and it showed good recovery and reliable accuracy and precision.

## Figures and Tables

**Table 1:** The results of the reproducibility study of the HPLC-CL with electrolytic reduction.

UQ (μM)	Precision (RSD, %)	
	Intra-Day (*n* = 5)	Inter-Day (*n* = 5)
0.10	2.8	2.5
1.0	3.4	3.1
8.0	3.2	3.5

**Table 2:** The results of the reproducibility study of the HPLC-CL with reduction column.

UQ (μM)	Precision (RSD, %)	
	Intra-Day (*n* = 5)	Inter-Day (*n* = 5)
0.10	2.8	4.4
1.0	2.9	3.1
8.0	2.9	1.9

**Table 3:** The comparison between the proposed methods and the previous DTT method.

	Electrolytic Reduction	Reduction Column	DTT Method [25]
* LOD (μM)	0.0031	0.0045	0.030
** *N*	6618	3975	2405
Pump	2 pumps	2 pumps	3 pumps

* Limit of detection. ** Number of theoretical plates.

**Table 4:** The recovery and precision of UQ determination in spiked human plasma employing the proposed electrolytic reduction method.

UQ (μM)	Recovery (%)	Precision (RSD, %)
0.10	111	4.0
0.50	97.9	6.8
2.0	108	4.3

**Table 5:** The comparison of the sensitivity of the proposed methods with the previously reported methods in the literature.

Method	* LOD (nM)	LOD (pmol/ Injection)	Application	Ref.
Spectrophotometry	286	―	Pharmaceutics/plasma	[15]
^1^H-NMR	9030	―	Food supplements	[17]
HPLC-UV	20	0.040	Human plasma	[18]
HPLC-ECD	5.8	0.11	Human plasma	[2]
HPLC-ECD	2.5	0.050	Human plasma	[3]
HPLC-ECD	17	0.34	Mouse tissues (liver, heart, muscle, brain)	[19]
LC-MS/MS	6.4	0.30	Human serum	[6]
HPLC-DTT-CL	30	0.16		[25]
HPLC-Electrolytic reduction-CL	3.1	0.016	Human plasma	This method

* Limit of detection.

## Data Availability

The data will be available upon reasonable request.
